# Combined antioxidant effects of Neem extract, bacteria, red blood cells and Lysozyme: possible relation to periodontal disease

**DOI:** 10.1186/s12906-017-1900-3

**Published:** 2017-08-10

**Authors:** Leali Heyman, Yael Houri-Haddad, Samuel N. Heyman, Isaac Ginsburg, Yossi Gleitman, Osnat Feuerstein

**Affiliations:** 1 Department of Prosthodontics, Hebrew University-Hadassah Faculty of Dental Medicine, P.O.B. 12272, 91120 Jerusalem, Israel; 2 Department of Medicine, Hadassah-Hebrew University Hospital, Mount Scopus, Jerusalem, Israel; 3 Institute for Dental Research, Hebrew University-Hadassah Faculty of Dental Medicine, Jerusalem, Israel

**Keywords:** Plants, Polyphenols, *Porphyromonas gingivalis*, Inflammation

## Abstract

**Background:**

The common usage of chewing sticks prepared from Neem tree (*Azadirachta indica*) in India suggests its potential efficacy in periodontal diseases. The objective of this study is to explore the antibacterial effects of Neem leaf extract on the periodontophatic bacteria *Porphyromonas gingivalis* and *Fusobacterium nucleatum*, and its antioxidant capacities alone and in combination with bacteria and polycationic peptides that may be at the site of inflammation.

**Methods:**

Neem leaf extract was prepared by ethanol extraction. The growth kinetics of *P. gingivalis* and *F. nucleatum* under anaerobic conditions in the presence of Neem leaf extract were measured. Broth microdilution test was used to determine the Minimal Inhibitory Concentration (MIC) of Neem leaf extract against each bacterial strain. The effect of Neem leaf extract on the coaggregation of the bacteria was assessed by a visual semi-quantitative assay. The antioxidant capacities of Neem leaf extract alone and in combination with bacteria, with the addition of red blood cells or the polycationic peptides chlorhexidine and lisozyme, were determined using a chemiluminescence assay.

**Results:**

Neem leaf extract showed prominent dose-dependent antibacterial activity against *P. gingivalis*, however, had no effect on the growth of *F. nucleatum* nor on the coaggregation of the two bacteria. Yet, it showed intense antioxidant activity, which was amplified following adherence to bacteria and with the addition of red blood cells or the polycationic peptides.

**Conclusions:**

Neem leaf extract, containing polyphenols that adhere to oral surfaces, have the potential to provide long-lasting antibacterial as well as synergic antioxidant activities when in complex with bacteria, red blood cells and lisozyme. Thus, it might be especially effective in periodontal diseases.

## Background

Periodontitis is among the most prevalent oral diseases. It is a multifactorial polymicrobial infection, induced primarily by oral anaerobic bacteria such as *Porphyromonas gingivalis* and *Fusobacterium nucleatum*. The adherence of bacteria to mucosal cells is a critical step in the development of periodontal infection. *P. gingivalis* is able to adhere to cellular and acellular surfaces [1], form a biofilm [2], and function as a keystone pathogen, by elevating the virulence of the entire microbial community and interfering with host immunity [3]. *F. nucleatum* is capable of invading epithelial cells [4], stimulating proinflammatory cytokine expression [5], and expressing a variety of surface adhesins, allowing coaggregation with most oral bacteria [6, 7]. As *P. gingivalis* and *F. nucleatum* are strongly associated with periodontitis, their suppression may be important in controlling the disease.

The Neem tree (*Azadirachta indica*), an evergreen, is a member of the Mahogany family *Meliaceae*. It is a native plant in the subcontinent of India and Myanmar that has been widely planted and naturalized in semi-arid areas throughout Asia and Africa [8, 9]. Neem extracts are popular folk medicines in India [8–10]. Extracts, produced from various parts of the tree were found to contain a diverse selection of polyphenols (e.g. tannins, lignins, flavanoids) possessing strong antioxidant [8, 9, 11], antibacterial [10–15], as well as anti-inflammatory and immunomodulatory properties [16–20].

The beneficial use of chewing sticks made of Neem tree twigs in India [8], as recommended by the 2000 World Health Organization (WHO) Consensus Report on Oral Hygiene [21], might be related to the mechanical act of plaque removal itself and enhanced salivation [22]. However, it is conceivable that the antibacterial and antioxidant components in these chewing sticks also play a role. The influence of aqueous Neem preparations on oral streptococci has been explored in several in vitro studies [13–15]. An aqueous Neem bark preparation inhibited oral streptococci and induced a significant reduction in their surface adhesion [14]. Indeed, a significant decrease in plaque accumulation and bacterial counts were found following oral treatment with Neem extract [23]. However, to the best of our knowledge, no studies were performed on the possible beneficial effects of Neem extracts on the microbiota involved in periodontitis [24–27].

In addition to their antimicrobial capacity, Neem extracts possess potent anti-inflammatory [28] and antioxidant properties [8, 9], which may suppress oxidative stress that accompany periodontal pathologies. Thus, one may assume that in addition to their antibacterial activity, Neem extracts may ameliorate periodontal diseases by the attenuation of inflammation through the suppression of reactive oxygen species (ROS), exerted by the plant-derived antioxidants. Furthermore, recent studies have shown that a variety of microbial species, platelets and red blood cells (RBC) have the ability to avidly bind to their surfaces a large assortment of plant-derived antioxidant flavonoids [29, 30], and such coated cells show marked antioxidative capacities. Polycationic peptides, such as lysozyme and chlorhexidine, which may be at the site of inflammation, also enhance the binding of polyphenols to bacterial surfaces, amplifying their antioxidant capacities [31].

The aim of this study was to explore the antibacterial effects of Neem leaf extract on the periopathogens *P. gingivalis* and *F. nucleatum*, and to evaluate its antioxidant capacities alone, and in complexes with bacteria, with polycationic peptides or with RBC, which are often found in the environment of inflamed periodontal tissues.

## Methods

### Preparation of Neem leaf extract

Fresh leaves of Neem tree, *Azadirachta indica* A. Juss (Meliaceae) [32], with a voucher specimen (#1611) that was deposited in the herbarium of the Center for Sustainable Agriculture, were identified and supplied by Dr. Solowey (The Arava Institute for Environmental Studies, Kibbutz Ktora, Israel). Neem leaves were dried, ground and suspended in ethanol (30%) for 2 months at a concentration of 0.24% (*w*/*v*) [32]. Following centrifugation (5000 RPM, 10 min) the supernatant, containing the extracted Neem stock solution, was kept at 22 °C in an opaque flask. The concentrations of polyphenols in the Neem leaf extracts were expressed as μM of Gallic-Acid Equivalents (GAE), using the Folin-Ciocalteu method [33].

### Microorganisms and growth kinetic conditions

*P. gingivalis* (ATCC 33277) and *F. nucleatum* (ATCC 1924) were grown in Wilkins-Chalgren anaerobic broth (Oxoid Ltd., Basingstoke, Hampshire, England) under anaerobic conditions of 5% CO_2_, 10% H_2_, and 85% N_2_ at 37 °C (Coy anaerobic chamber). All bacteria were subcultured twice and were grown to the early stationary phase. The final concentration of *P. gingivalis* and *F. nucleatum* was adjusted to 1.5X10^11^ bacteria/ml and 0.5X10^11^ bacteria/ml, respectively (OD_650nm_) Bacterial growth kinetics were determined at different concentrations of Neem leaf extract using a Thermomax-automated microtiter plate reader (Molecular Devices Corp, Sunnyvale, CA). Optical density measurements were performed periodically under aerobic conditions at 37 °C throughout the incubation period (24 h and 48 h, for *F. nucleatum* and *P. gingivalis*, respectively).

### Broth microdilution MIC (minimal inhibitory concentration) test

Broth microdilution was performed in 96-well plate with Wilkins-Chalgren anaerobic broth. Twofold dilutions of Neem leaf extracts ranging from final concentration of 20 to 0.039 μM GAE were used. A positive control of standard antibiotic for anaerobic infections therapy, metronidazole (Sigma), was prepared into final concentrations ranging from 2 to 0.004 μg/ml. The solvent used (30% ethanol) was served as a negative control. The standardized of each bacterial inoculum was adjusted to 0.5 McFarland standard (equivalent to 1.5 × 10^8^ CFU/ml) per well. The microtiter plates were incubated at 37 °C under anaerobic conditions of 5% CO_2_, 10% H_2_, and 85% N_2_ at 37 °C (Coy anaerobic chamber). MICs were read after 72 h of incubation at OD_650nm_ using Thermomax-automated microtiter plate reader (Molecular Devices Corp, Sunnyvale, CA). The MIC test was repeated 2 times in duplicates (*n* = 6).

### Coaggregation visual assay

Bacterial coaggregation confers virulence and facilitates the generation of plaque [6, 7, 34]. Therefore, in addition to the assessment of bacterial growth, studies were also conducted to determine the impact of Neem leaf extract upon bacterial co-aggregation. Coaggregation of the *P. gingivalis* and *F. nucleatum* strains was assessed in the absence and presence of Neem leaf extract, by means of a visual semi-quantitative assay [34]. The bacterial strains were grown as described above, and harvested at the late exponential or early stationary phase. The strains were washed in coaggregation buffer (CAB) containing: 0.1 mM CaCl_2_, 0.1 mM MgCl_2_ 0.15 M NaCl and 0.02% NaN_3_, dissolved in 1.0 mM Tris, pH 8.0, and resuspended to OD_660nm_ = 1.0. Equal volumes of 150 μl of each bacterial suspension (*F. nucleatum* and *P. gingivalis*) were mixed in test tubes by vortex with the addition of 150 μl of Neem leaf extract or PBS (control). Coaggregation was scored according to a 0 to 4 scale, as described by Cisar et al. [34]: 0**-**no visible aggregates, with unaltered turbidity; 1-finely dispersed aggregates in a turbid solution; 2-presence of definite aggregates which did not settle immediately; 3-large settling aggregates but with slightly turbid supernatant; and 4-formation of large, fast-settling aggregates, with the supernatant remaining water-clear.

### Determination of the oxidant scavenging abilities (OSA) of Neem leaf extract by a Luminol-dependent Chemiluminescence (LDCL) assay

The OSA of Neem leaf extract was determined using a LDCL-generating cocktail containing luminol (10 μM), glucose oxidase (GO) (23 U/mg dry weight), sodium selenite [IV] (100 μM) and 10 μM Co [II] [35, 36]. The light emitted was due to the generation of superoxide, H_2_O_2_, and the hydroxyl radical. Light quenching was measured at 22 °C with a LUMAC 2500 M Biocounter (Landgraaf, Netherlands) and expressed as counts per minute (cpm). Luminescence was recorded at 30 s intervals for up to 5 min. The OSA of the Neem leaf extract tested was evaluated by its ability to quench the light generated by the cocktail. Catechin, a natural polyphenol from tea with confirmed antioxidant properties, served as a positive control.

### Determination of OSA resulting from the interaction between Neem leaf extract, bacteria, red blood cells (RBC) and polycationic peptides

Neem leaf extract may bind to mammalian and microbial surfaces [29, 30, 37–39], which can affect OSA. Various amounts of neem leaf extract were added to microbial suspensions, incubated for 10 min at room temperature, washed several times to remove unbound agents, and then re-suspended in 800 μl Hank’s balanced salt solution (HBSS). Fresh heparinized human blood, obtained by consent (Approval HMO 0313–11, Helsinki Committee Board of the Hadassah Medical Center, Jerusalem, Israel), was washed several times in normal saline and the pelleted RBC were then re-suspended in HBSS. RBC or the polycationic peptides lysozyme (found in saliva), 20 μl of 20 μg/ml, and chlorhexidine (used in commercial mouthwash solutions), 20 μl of 0.2% solution, were added to microbial suspensions with or without co-adsorption of Neem extract polyphenols. The OSA resulting from the interaction between the bacteria cells coated with Neem leaf extract and RBC or polycationic peptides were determined by luminescence (see above).

### Statistical analysis

The results were expressed as the mean ± SEM. One or two-way ANOVA with the Newman-Keuls test for post-hoc comparisons were applied. Differences were considered statistically significant at *p* < 0.05.

## Results

### The antibacterial activity of Neem leaf extract

**Fig. 1:**
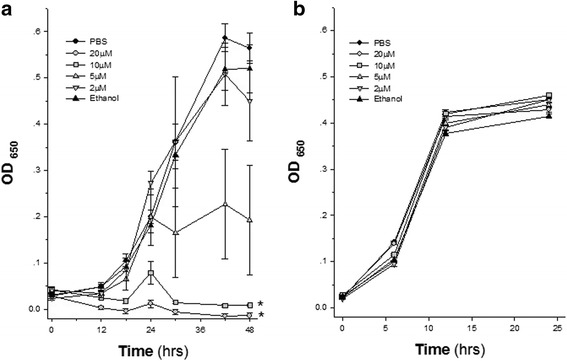
Bacterial growth kinetics of *P. gingivalis* (**a**) and *F. nucleatum* (**b**) during 48 h and 24 h incubation, respectively. Six experimental groups were compared for each bacterial strain: two control groups (Neem-free bacterial suspensions, containing ethanol or PBS), and six Neem leaf extract groups with increasing concentrations, expressed as Gallic Acid Equivalent (GAE). *n* = 6 for each experimental group. * *P* < 0.005, two-way ANOVA vs. PBS and ethanol groups

Figure 1a shows the effect of increasing concentrations of Neem leaf extract (expressed as GAE) on *P. gingivalis* growth. Whereas approximately 50% growth inhibition was induced by 5 μM GAE of Neem leaf extract, higher concentrations (10 and 20 μM GAE) markedly inhibited *P. gingivalis* growth (*p* < 0.005, two way ANOVA). Significant growth inhibition was noted already after 18 h (*p* < 0.05, one way ANOVA). In contrast, Neem leaf extract had no effect on the growth of *F. nucleatum* even at the highest concentrations (Fig. 1b).

Determining the MIC using broth microdilution test enabled a better assessment of the two bacterial strains sensitivity to Neem leaf extract at extensive range of concentrations. MIC value, the lowest concentration of Neem leaf extract at which there was no visible growth of the organism, was defined as 10 μM GAE for *P. gingivalis*. However, *F. nucleatum* showed resistance to all tested dilutions of Neem leaf extract. The MIC values for the positive control, metronidazole, were within the expected MIC [40], between 0.016 and 0.031 μg/ml for *P. gingivalis* and 0.063 and 0.125 μg/ml for *F. nucleatum.*

### Coaggregation visual assay

Coaggregation of *P. gingivalis* and *F. nucleatum* in the presence of Neem leaf extract was assessed using a coaggregation buffer. Both the control and samples treated with Neem leaf extract caused the formation of large precipitating aggregates and a slightly turbid supernatant, compatible with a Cisar scale grade of 3 (data not shown) [34]. This indicated that Neem leaf extract had no influence on the coaggregation of *P. gingivalis* and *F. nucleatum*.

### Effect of Neem leaf extract and bacteria coated by Neem leaf extract on OSA, determined by LDCL assay

**Fig. 2:**
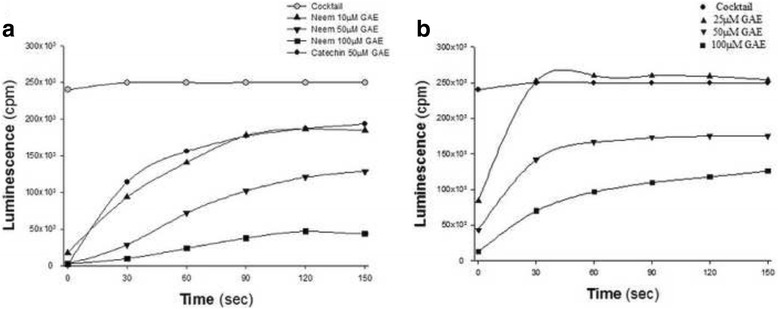
Modulation of luminescence by Neem leaf extract. Total oxidants, generated by the cocktail, were measured by luminescence. A time-dependent chemiluminescence pattern of oxidant scavenging abilities (OSA) induced by: (**a**) increasing concentrations of Neem leaf extract expressed as Gallic Acid Equivalnce (GAE): 10 μM, 50 μM, and 100 μM. Catechin (50 μM GAE), a natural polyphenol from tea with confirmed antioxidant properties, served as a positive control. **b** *P. gingivalis* pre-coated with increasing amounts of Neem leaf extract: 25 μM, 50 μM, and 100 μM GAE

As shown in Fig. 2a, Neem leaf extract dose-dependently induced the quenching of light, indicating its oxidant scavenging activity. The antioxidant capacity of Neem leaf extract (10 μM GAE), assessed with the luminescence assay, was roughly equivalent to that of catechin (50 μM GAE), a natural polyphenol with confirmed antioxidant properties. Figure 2b shows the dose-dependent OSA resulting from the effect of *P. gingivalis* pre-coated with Neem leaf extract. It should be noted that *P. gingivalis* alone did not possess any OSA.

### The OSA resulting from combinations between bacteria cells coated with Neem leaf extract and RBC or polycationic peptides

**Fig. 3:**
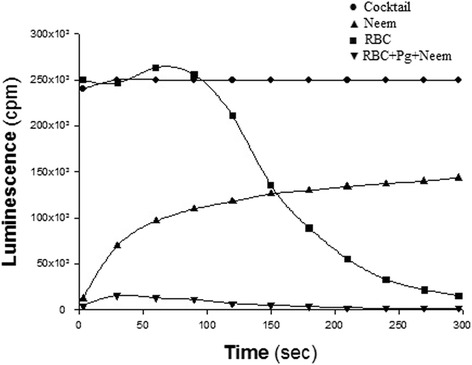
Modulation of luminescence by red blood cells (RBC) and complexes of *P. gingivalis* pre-coated with Neem leaf extract. Total oxidants generated by the cocktail, were measured along time by luminescence. Time-dependent chemiluminescence patterns of oxidant scavenging abilities (OSA) induced by *P. gingivalis* pre-coated with Neem (50 μM GAE), RBC alone, and combinations of RBC and *P. gingivalis* pre-coated with Neem

As shown in Fig. 3, RBC alone induced a typical U-shaped pattern of light scavenging, resulting from the gradual intracellular diffusion of the ROS and their intracellular decomposition, mainly by catalase. The OSA of *P. gingivalis* coated with Neem leaf extract was further markedly enhanced by small amounts of RBC.

**Fig. 4:**
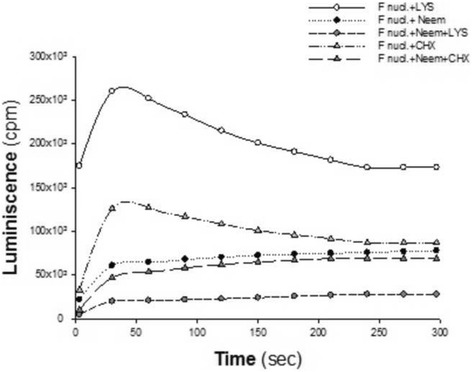
Modulation of luminescence by *F. nucleatum* alone or complexed with Neem leaf extract (50 μM GAE), when incubated with one of the following peptides: Lysozyme (LYS, 20 μl of 20 μg/ml) or chlorhexidine (CHX, 20 μl of 0.2% solution)

Similarly, as shown in Fig. 4, *F. nucleatum*-Neem-lysozyme complex exerts greater OSA, with about 85% reduction of luminescence, as compared to *F. nucleatum-*Neem complex. However, the addition of the polycationic peptide chlorhexidene to *F. nucleatum*-Neem complex did not intensify the antioxidant effect of *F. nucleatum*-Neem complex, alone.

## Discussion

Our results show that an ethanol extract derived from Neem tree (*Azadirachta indica*) leaves has antibacterial properties against the periodontophatic bacterium *P. gingivalis* and potent antioxidant abilities. Our findings also show that small numbers of RBC further enhance the OSA of *P. gingivalis* pre-coated with agents present in Neem leaf extract, and that lysozyme also amplifies the OSA of bacteria pre-coated with Neem leaf extract.

Neem leaf extract has no effect on the coaggregation of *P. gingivalis* and *F. nucleatum*. Also, surprisingly, it has no effect on the growth of *F. nucleatum*, as opposed to its antibacterial effect on *P. gingivalis,* when tested under similar experimental conditions (Fig. 1), maybe due to differences in bacterial cell surfaces between different species, suggesting the contribution of species specific agents in the Neem leaf extract. Other studies using different polyphenolic extracts on *P. gingivalis* and *F. nucleatum* exhibited diverse effects, such as antibacterial activities against both bacteria by propolis extracts [41], and no effect on their growth and viability by cranberry juice [42]. On the other hand, in our previous study on a component of cranberry we showed that it inhibits bacterial coaggregation, affects biofilm formation and immune cells, and attenuates the severity of experimental periodontitis [43, 44]. These diverse activities of the different polyphenolic extracts could be explained by an altered mechanism of action of those compounds. Thus, a mixture of extracts from several natural substances, such as Neem and cranberry, appears to have the benefit of combining their dissimilar and complementary activities for more effective treatment of periodontitis. There is a growing interest in plant extracts and oils as alternative antimicrobial agents for treating various infections [45, 46].

Neem leaves are extremely rich in a large variety of polyphenol agents, possessing antioxidant properties that can modulate inflammation [16–18]. Furthermore, Neem polyphenols can avidly bind to surfaces of both microbial and mammalian cells and to endow them with potent antioxidant properties (Fig. 2). The results are similar to the effect shown for a large variety of plant extracts and reagent polyphenols, used to coat different microbial species [29, 30]. Therefore, bacteria coated with polyphenols seem to be an excellent mode for achieving long-lasting antioxidant activity in the oral mucosa, with the bacteria, alive or killed by the polyphenols, serving as carriers of these molecules. It is interesting that binding of Neem polyphenols to the cell surface might be further enhanced by RBC and salivary cationic lysozymes, which may act as adhesives. This amplification of antioxidant capacity bears clinical relevance as lysozyme is always present in the oral cavity, while RBC escape injured capillaries, in particular those adjacent to inflamed periodontal tissues. Moreover, one may assume that the presence in the oral cavity of additional cationic agents released from neutrophils may also increase the anti-inflammatory and antioxidant capacities of Neem extracts. Indeed, our findings illustrate that RBC and lysozyme might substantially intensify the antioxidant capacity provided by bacteria-Neem complex, conceivably by increasing bound polyphenols. The lack of intensification of OSA by the addition of another polycationic compound, chlorhexidine, to *F. nucleatum*-Neem complex, as compared to the antioxidant capacity of this complex alone deserves further evaluation.

Taken together, Neem extract, adsorbed by bacteria, in addition to its antibacterial properties, provides profound antioxidant activity, intensified in the presence of RBC or lysozyme. However, the potent antioxidant activity in Neem extracts might also act as a double-edged sword. On one hand scavenging ROS might lower inflammatory damage to the gum, but on the other hand it may also protect periopathogenic catalase-negative bacteria against oxidants generated by inflammation or cariogenic streptococci.

In addition, our report has two major limitations: First, the tested species in this study were evaluated in a planktonic state, whereas, in vivo they are part of a multi-specious biofilm, which has a profound impact in the effectiveness of antimicrobial agents. Therefore, further clinical studies using periodontal parameters are needed to shed more light on the potential clinical implication of Neem extracts as a modulator of periodontal diseases. Second, we studied fresh leaves obtained from Neem trees grown in Israel, applying a specific extraction protocol. Further experiments are needed to extend our findings to Neem extracts obtained from other parts of this plant and to explore comparable extracts produced from trees grown abroad. Furthermore, we did not label and quantify the numerous compounds plausibly extracted from the plant, and we did not identify components responsible for the antimicrobial properties of the extract. Nor did we compare the characteristics of our product with extracts obtained by other methods. Additional studies are needed to address these important limitations, and safety evaluation is required preceding clinical trials.

## Conclusions

We report that Neem extracts possess prominent dose-dependent bacteriostatic activity against *P. gingivalis* but not against *F. nucleatum*. It possesses strong anti-oxidant activity, which is amplified following adherence to bacteria, to red blood cells and to the polycationic peptide lysozyme. The clinical relevance of these findings is based on the co-existence of bacteria, RBCs and polycationic compounds in the oral cavity. Conceivably, Neem extract could adhere to these compounds, and provide a long lasting bacteriostatic as well as anti-oxidant activity at the site of inflammation in periodontal diseases. Prospective randomized trials in-vivo are needed to strengthen these in-vitro findings, suggesting potential clinical implication of the use of Neem leaf extract in periodontal diseases.

## Abbreviations

GAE: Gallic-acid equivalents
LDCL: Luminol-dependent chemiluminescence
OSA: Oxidant scavenging abilities
ROS: Reactive oxygen species
